# Postfire Scenarios Shape Dung Beetle Communities in the Orinoquía Riparian Forest–Savannah Transition

**DOI:** 10.3390/biology14040423

**Published:** 2025-04-15

**Authors:** Carlos Julián Moreno-Fonseca, Jorge Ari Noriega, Walter Garcia-Suabita, Dolors Armenteras-Pascual

**Affiliations:** 1 Grupo de Investigación en Ecología del Paisaje y Modelación de Ecosistemas-ECOLMOD, Departamento de Biología, Universidad Nacional de Colombia, Bogotá 111311, Colombia; walter.suabita@gmail.com (W.G.-S.); darmenterasp@unal.edu.co (D.A.-P.); 2 Grupo de Agua, Salud y Ambiente, Programa de Ingeniería Ambiental, Facultad de Ingeniería, Universidad El Bosque, Bogotá 111321, Colombia

**Keywords:** fire occurrence, time since the last fire event, resilience, dung beetle assemblages

## Abstract

The Orinoquía region of Colombia includes diverse ecosystems such as riparian forests and seasonal savannas, which play vital roles as biodiversity reservoirs. However, increased fire activity, driven by both natural and anthropogenic pressures, poses mounting threats to these ecosystems. Despite their importance, the effects of fire on faunal communities, especially in transitional habitats, are not well understood. Understanding biodiversity responses to fire across different recovery stages is essential for conservation planning. This study aimed to assess the effects of fire occurrence and recovery time on dung beetle communities as an indicator of ecosystem resilience. We analyzed taxonomic responses—including species richness, abundance, and Hill diversity indices (D_0_, D_1_, D_2_)—as well as functional traits such as guild richness, biomass, and food relocation behavior, across riparian forest–savanna ecotones under varying fire histories. Our results indicate that recent fires (≤1 year) and high fire frequencies (4–5 events) negatively affect species diversity and abundance. Dominance by a few disturbance-tolerant species, such as *Digitonthophagus gazella*, was observed in burned savannas, while forest habitats supported both rare and dominant taxa. Despite taxonomic declines, functional redundancy was maintained, largely due to the prevalence of small-bodied species. However, we observed a general resilience effect in which core species contributed to postfire community reassembly. Functional redundancy was maintained, with small dung beetles dominating the biomass and guild composition. The conservation status of transitional habitats, particularly the forest–savanna ecotone, played a critical role in postfire dung beetle community restructuring. The presence of resilient assemblages highlights the importance of dung beetles in sustaining key ecosystem functions following fire events. These findings underscore the potential of dung beetles as bioindicators for postfire monitoring and emphasize the need for improved fire management strategies in sensitive ecosystems.

## Simple Summary

Fire is a significant ecological force that alters ecosystem biodiversity and ecological processes. Understanding how fauna respond to fire is critical, particularly in ecosystems that serve as biodiversity reservoirs. The riparian forests and savannas of Orinoquía offer important ecosystem services, with their role as refuges for biodiversity, climate regulation and carbon sequestration being among the most important. Despite this, these ecosystems are increasingly disrupted by fire. Dung beetles are recognized as bioindicators of habitat disturbance due to their sensitivity to environmental changes and their ecological role in dung recycling, secondary seed dispersion and soil removal. This study investigates the response of dung beetle communities to fire across various conservation settings within riparian forest–savanna transition zones. Our results showed that recent fires had a detrimental effect on the composition and diversity of dung beetles. However, ecosystems that remained unburned or had undergone long-term recovery (10–16 years postfire) had a critical function in restructuring dung beetle communities. These results offer insights on the role of specific habitat community structure in postfire ecological dynamics and enhance the use of dung beetles taxonomic and functional assemblage descriptors in monitoring ecosystem recovery.

## 1. Introduction

Fire represents a natural disturbance that induces functional and structural changes in ecosystems. The various impacts that can be sustained by natural systems depend on the interaction of spatial and temporal scales [1]. In this context, the potential effects of fires on fauna and the subsequent responses to postfire scenarios are influenced by the faunistic group analyzed, the biogeographical area, and the characteristics of the fire regime [2,3]. Information from sensitive tropical ecosystems that are affected by fire points out that, while there is no general pattern of faunal responses to fire, including more descriptor variables of fires can improve understanding of the ecological process and explain the differential responses of different faunal communities [4].

The riparian forests of the Colombian Orinoquía serve as crucial biodiversity reservoirs, providing ecological connectivity between piedmont ecosystems and the Amazonian rainforest [5,6]. These forests, characterized by their linear structures along watercourses, are embedded within seasonal and hyperseasonal savannas [7]. However, human activities such as agriculture, hunting, and logging have significantly altered land use in these landscapes, with fire becoming a commonly used tool for land management [8]. During drought seasons, fires frequently escape control because of accumulated fuel loads, low humidity, and strong winds [9]. The increasing frequency and intensity of wildfires, exacerbated by climate change and extreme weather events such as the El Niño–Southern Oscillation (ENSO), further threaten biodiversity [1,10]. In this sense, extensive and persistent droughts alter fire regimes, negatively impacting—both directly and indirectly—the survival, reproduction, and dispersal of fire-sensitive species, thereby reducing their potential capacity for recovery and persistence after a fire [11,12,13].

The ecological relationship between fire and biodiversity is shaped by fire-induced disturbances that alter forest structure [14,15,16]. These structural changes trigger cascading effects on faunal communities, impacting species abundance, functional roles, and, ultimately, ecosystem stability [1,17]. In tropical forest–savanna transition zones, fires strongly influence landscapes [18,19]. However, the structural characteristics of Orinoquía’s riparian forests resemble those of tropical rainforests, making them particularly fire sensitive [20,21].

The degree to which fauna is affected depends on species-specific adaptations and fire characteristics [12]. Some species thrive in postfire environments because of their opportunistic traits (e.g., dispersion capacity, access to exposed resources, thermal tolerance, polivoltinism), whereas others, particularly those sensitive to microclimate changes, suffer population declines [22,23]. Unburned habitat patches play a vital role in maintaining populations of both fire-sensitive and fire-adapted species by offering refugia and resource stability [24,25,26,27]. Fire frequency, occurrence, and time since the last fire event (TSLF) are key determinants of biodiversity responses, shaping ecological succession and species recovery patterns [4,28].

This study evaluated the effects of fire on dung beetle communities in the riparian forest–savanna transitions of the Orinoquía region. Dung beetles (Coleoptera: Scarabaeidae) serve as effective bioindicators of habitat disturbance because of their strong responses to environmental changes and their essential roles in ecosystem processes, including dung removal, nutrient recycling, soil aeration (bioturbation), secondary seed dispersal, and parasite control [29,30,31,32,33,34]. Their functional traits, particularly those linked to body size and feeding strategies (e.g., biomass, food relocation, guild structure), offer measurable insights into ecosystem resilience following fire disturbances [35,36]. The effects of fire on beetles have been variable, with some specific patterns identified at the level of fire-sensitive ecosystems. Richness and abundance are variables that respond in contrasting ways, where in some cases resilient communities dominated by species adapted to open environments are identified [37,38,39], while in assemblages with a greater presence of stenotopic species, these variables can be seen to be reduced by the effects of fire [40,41,42]. Although the potential effects of fire on the functional aspects of dung beetles have been poorly evaluated, a diminishing effect has been recognized in some variables such as biomass, dung removal, and seed dispersal [43,44]. In most cases, fire descriptors have been included indirectly where fire occurrence has been the aspect generally evaluated. In this regard, it is necessary to recognize beetle responses in different postfire scenarios (more than 2 years) since this information is vitally important for recognizing the ecological recovery processes from these disturbances and is even more so in fire-sensitive ecosystems [4]. Understanding these effects on dung beetles is relevant not only because these organisms act as a keystone in ecological processes but also helps elucidate their importance as a monitoring element for the conservation of ecosystems impacted by fire.

We assessed the dung beetle community responses to different fire scenarios (fire occurrence and TSLF) across two conservation settings. Our study aimed to answer the following questions: (1) How do dung beetle composition, richness, abundance (taxonomic and functional), and biomass vary under different fire scenarios? (2) How do specific habitats of the riparian forest–savannah transition influence responses to fire at the taxonomic and functional levels? (3) What is the relationship between conservation status and fire effects on dung beetle assemblages?

Understanding these patterns is crucial for developing conservation strategies that incorporate fire as a key ecological driver while mitigating its adverse effects in fire-sensitive ecosystems.

## 2. Materials and Methods

### 2.1. Study Sites and Dung Beetle Sampling

**Figure 1:**
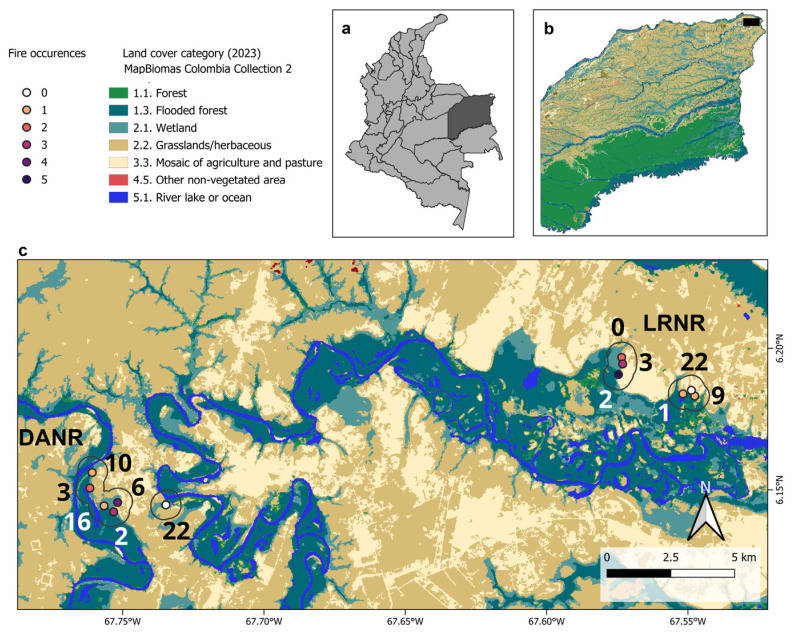
Locations of the sampling sites in the Doña Ana Natural Reserve (DANR) and Los Robles Natural Reserve (LRNR). With reference to Colombia (**a**), the Vichada department (**b**), and the Puerto Carreño Landscape (**c**). The numbers next to the sampling sites indicate the number of years since the last fire event (TSLF).

The study was conducted at two private natural reserves, Doña Ana (DANR) (06°08′32.4″ N–67°45′07.8″ W) and Los Robles (LRNR) (06°10′59.5″ N–67°32′48.9″ W), located in Puerto Carreño within the Orinoquía region (Figure 1). Both reserves comprise a mosaic of natural savannas and riparian forests that are subject to seasonal fire and flood dynamics. Fire events primarily occur during the dry season (December–March), whereas flooding peaks during the rainy season (April–October). DANR is strictly protected, with prohibitions on hunting and illegal logging, which promotes habitat conservation for local fauna. On the other hand, LRNR faces continuous anthropogenic pressures, including illegal wood thinning, poaching, and frequent burning. Collections were made in at 36 sample points (18 per reserve, categorized into three habitat types): savanna (6 sites per reserve), forest–savanna edge (6 sites per reserve), and forest interior (6 sites per reserve). Fire data for each location were obtained via the Fire Information for Resource Management System (FIRMS) (data accession date: 11 October 2024), which provides near-real-time active fire hotspot data via Moderate Resolution Imaging Spectroradiometer (MODIS) sensors aboard the Aqua and Terra satellites. We recorded fire occurrence (FOc: 0–5 events) and time since the last fire event (TSLF: 0–23 years) within a 0.5 km buffer around each site (Table S1).

Field work was conducted during the dry seasons of March–April 2021 and 2022, ensuring standardized climatic conditions and minimizing seasonal effects on dung beetle assemblages. To maintain sample independence, the locations were spaced ≥ 0.5 km apart [45]. At each location, we placed a linear transect (150 m) consisting of three pitfall traps spaced 50 m apart, each arranged in a savanna, edge, or interior forest site. All traps were baited with 40 g of a 1:1 human and pig dung mixture and remained active for 24 h at each site. The pitfall traps consisted of plastic containers (diameter 16 cm and depth 9 cm) installed at ground level, which were partly filled with 250 mL of 70% ethanol. The baits were placed on a cotton mesh at the center of each trap, with a wire as a bait holder. Each trap had a plastic lid that was 15 cm in diameter and was suspended 20 cm above the surface to avoid the effects of desiccation, rain, and animal damage.

### 2.2. Functional Trait Characterization

Dung beetles were categorized on the basis of four functional traits relevant to ecosystem processes:

#### 2.2.1. Biomass

A subset of 1–10 individuals per species was oven-dried at 40 ± 5 °C until a stable weight was reached and weighed with a precision scale (±0.0001 g accuracy) [45]. We conducted a comprehensive characterization of the biomass composition of dung beetle assemblages by employing community weighted means (CWMs), utilizing traits defined as the proportion of individuals classified within each food relocation category.

#### 2.2.2. Food Relocation

The categorization of food relocation behavior for each species was performed by field observations. According to the literature, the classification included the following categories: (1) rollers—species that arrive at the dung and remove portions, which are rolled horizontally and then buried; (2) tunnellers—species that bury portions of dung in tunnels vertically or adjacent to the site where the dung was originally deposited and transport dung to the bottom; and (3) dwellers—species that feed, live and nest inside the dung [46].

#### 2.2.3. Guilds

The size of dung beetles, measured by body weight, has been linked to their contributions to specific ecological functions at the individual, population, or community level. In this context, studies have shown that larger beetles tend to disperse larger seeds, and remove more excrement and soil compared to smaller ones [47,48,49,50]. In contrast, smaller beetles are associated with quicker access to and utilization of food resources and smaller seed dispersion [51,52,53,54,55]. This, along with the relocation strategies exhibited by dung beetles, helps define guilds not only based on how they access food resources, but also on the functional contributions that these groups of organisms can potentially make. On the basis of food relocation, weight, and size, we used a guild classification according to the literature [35,36]: small (≤20 mg), medium (21–100 mg), and large (≥101 mg) (rollers, tunnellers and dwellers).

#### 2.2.4. Habitat Specificity

For habitat specificity, species collected only from forests or savannas were considered specialists, whereas edge species were considered habitat generalists [56].

### 2.3. Data Analysis

#### 2.3.1. Species Richness and Diversity

Sampling sufficiency was assessed via coverage estimation [56], which was performed via the iNEXT package [57]. Taxonomic diversity was analyzed via Hill numbers (qD), which were calculated for three orders: (a) 0D (species richness), which is insensitive to abundance and thus assigns disproportionate significance to rare species; (b) 1D (Exponential Shannon entropy), which allocates weights to each species in accordance with its abundance within the community without preferential treatment toward either rare or abundant species; and (c) 2D (inverse Simpson concentration), which quantifies the number of very abundant or ‘dominant’ species present in the community [58,59].

#### 2.3.2. Beta Diversity and Bioindicator Analysis

To examine alterations in species composition in relation to various habitat types, TSVFs, and fire occurrence, a nonmetric multidimensional scaling (NMDS) ordination was conducted [60], utilizing a Bray–Curtis dissimilarity matrix of abundance with the basic R package (1.1.10) [61]. Furthermore, analysis of similarities (ANOSIM) was employed to evaluate the statistical significance of the associations between habitat type, TSLF, fire occurrence, and species similarity [62].

Indicator species analysis [63,64] was conducted to identify species that exhibit associations with distinct habitat types, TSLF and fire occurrence, predicated on their relative abundance and frequency, as well as to evaluate the magnitude of such associations. The ISA elucidates which species serve as indicators for various habitats while considering their specificity (operationally defined as the likelihood that a sample containing the species originates from a specified habitat type) and fidelity (defined as the likelihood that a species is sourced from a particular habitat type). Consequently, each species is allocated an indicator value (IV) that spans a continuum from 0 to 1 (calculated as the square root of the product of specificity and fidelity) [63]. Only the species that exhibited a statistically significant IV (assessed following 999 permutations; α = 0.05) were included in the resulting data.

#### 2.3.3. Functional Traits

Generalized linear mixed models (GLMMs) were used to assess biomass responses to fire variables (fire occurrence, TSLF) via the glmmTMB package (1.1.10) in R [65,66]. To ascertain the most appropriate structure for the fixed and random effects, we adopted a top-down approach that incorporated ‘habitat’, ‘TSLF’ and ‘fire occurrence’ as fixed factors and ‘location’ as a random factor. Biomass distribution across guilds was visualized via boxplots and assessed via the Kruskal–Wallis test.

## 3. Results

### 3.1. Dung Beetle Composition, Richness and Abundance (Taxonomic and Functional) Under Different Fire Scenarios

A total of 25.768 individuals belonging to 32 species were collected. Species richness was greater in the DANR (28 species) than in the LRNR (22.5 species, on average). The most abundant species were *Uroxys* cf. *brevis* (42.4%), followed by *Canthon juvencus* (5%) and *Atenoipsis* cf. *regulus* (4%), which are associated with unburned forests. *Digitonthophagus gazella* dominated savannah and border habitats with recent fire events (0–2 TSLF). Indicator species analysis identified *D. gazella* as a strong indicator of recently burned habitats (IndVal B = 0.833; *p* = 0.003) (Table S2). With respect to fire occurrence, *Dichotomius nisus* (IndVal B = 0.833; *p* = 0.009) and *Uroxys* cf. *brevis* (IndVal B = 0.833; *p* = 0.001) were identified as indicator species of sites with four and five fire occurrences, respectively (Table S1).

Species richness and abundance declined with increasing fire frequency and shorter recovery times, particularly in the LRNR. Recent fires (TSLF ≤ 1 year) and frequent fires (4–5 events) led to the lowest overall abundance and diversity. Particularly, the LRNR, dung beetle abundance declined significantly with increasing fire frequency, with the highest abundance in unburned forests and forests with TSLF = 2 years (ANOVA F = 9.111; *p* = 0.018). The average community abundance was 13.72 individuals in habitats with less than 1 year since the last fire event; in the unburned forest, the number of individuals was stable (prom = 231.6 individuals). Specifically, the transition from unburned forest to edge with 16 TSLFs and one fire occurrence had the highest abundance values (ANOVA F = 7.908; *p* = 0.001) (Figure S1).

**Figure 2:**
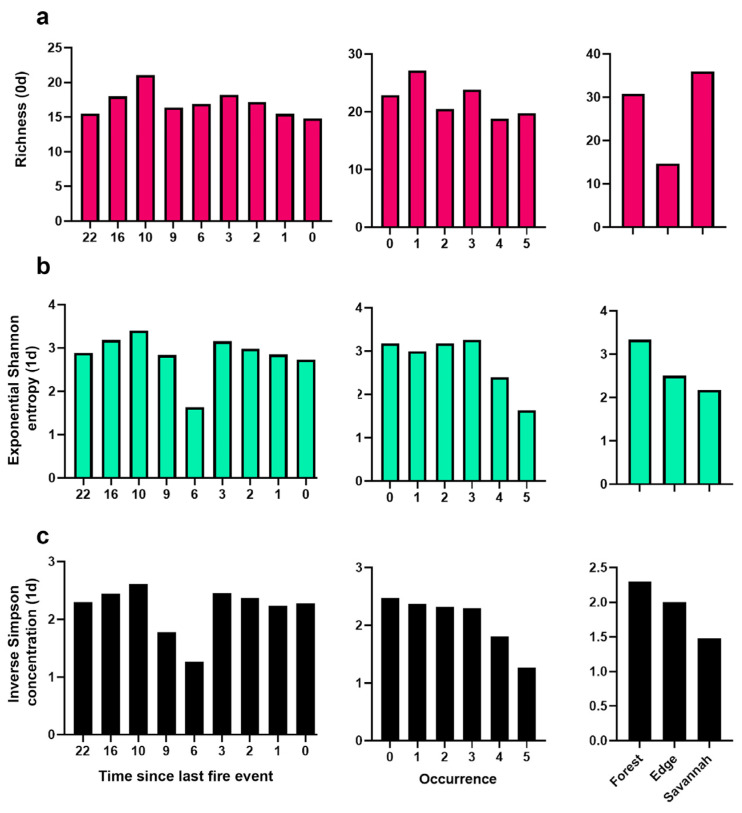
Abundance and taxonomic diversity (Hill numbers) of dung beetle assemblages across fire scenarios (occurrence and time since the last fire event (TSLF)) in forest, edge and savanna habitats. 0D richness—insensitive to abundance thereby giving a disproportionate weight to rare species (**a**), 1D exponential Shannon entropy (**b**), and 2D inverse Simpson concentration (**c**).

**Figure 3:**
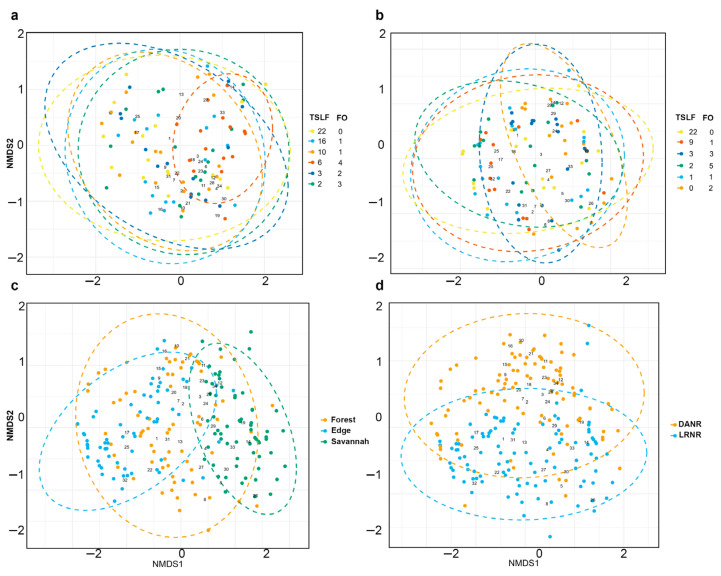
NMDS based on dung beetle assemblages according to fire occurrence and time since the last fire event (TSLF) in DANR (**a**) and LRNR (**b**), habitat (**c**), and between localities (DANR and LRNR) (**d**). FO: fire occurrence. For references to the numbers associated with species, see Table S1 in the Supplementary Materials.

Richness remained constant in terms of fire occurrence and time since the last fire scenario, with the highest values found in the edge habitat (Sprom = 5.36). Overall, species diversity was lower in sites with a TSLF = 6 years and in areas that had experienced 4–5 fire occurrences than in the other sampled sites (Figure 2c). A similar trend was observed for the dominant species, with additional declines recorded at sites with a TSLF = 9 years (Figure 2c). In contrast, ANOSIM revealed that both fire occurrences (R = 0.081; *p* = 0.002) and TSLF (R = 0.066; *p* = 0.005) had significant but weak influences on species similarity patterns. However, the NMDS analysis did not reveal clear segregation between the different fire scenarios (Figure 3a,b). Notably, communities from unburned habitats, particularly at forest edges, presented the largest cluster circumferences, encompassing the most fire-affected scenarios (Figure 3a,b).

**Figure 4:**
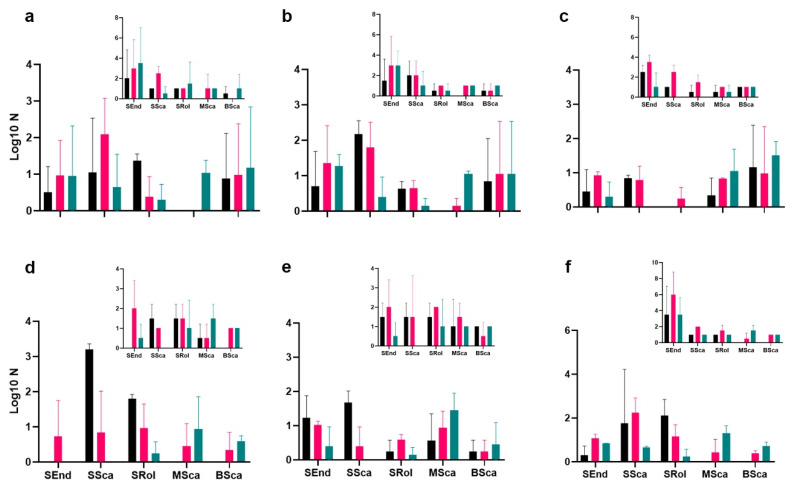
Dung beetle guild, abundance, and richness. Unburned habitats (DANR (**a**) and LRNR (**d**)); most recent fire scenario habitats (DANR (**b**) and LRNR (**e**)); and the highest number of fire occurrence habitats (DANR (**c**) and LRNR (**f**)). The inner figure corresponds to guild richness for each scenario. SEnd: small dwellers, SSca: small tunnellers, SRol: small rollers, MSca: medium-sized tunnellers, and BSca: large tunnellers; black: forest, red: edge, and blue: savannah.

**Figure 5:**
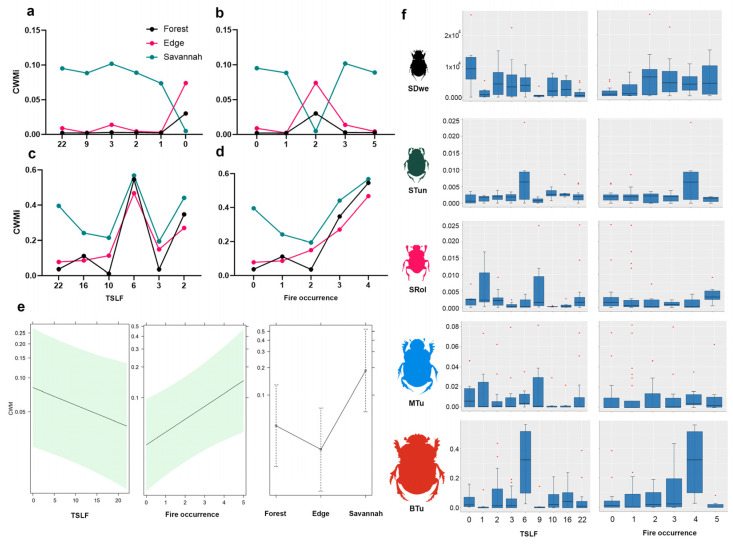
Total CWM according to TSLF (LRNR (**a**); DANR (**c**)) and fire occurrence (LRNR (**b**); DANR (**d**)). General linear mixed model (GLMM) of biomass (CWM) according to TSLF and fire occurrence (**e**). Dung beetle guild biomass (CWM) in fire scenarios. The green section refers to the 95% confidence interval (**f**). SDwe: small dwellers, STun: small tunnellers, SRol: small rollers, MTu: medium-sized tunnellers, and BTun: large tunnellers.

In terms of functionality, the dung beetle guild structure varied across fire scenarios, with small tunnellers dominating forest and edge habitats in both reserves, irrespective of fire occurrence and TSLF (Figure 4). However, their abundance declined sharply in habitats with recent fire outbreaks (TSLF = 0 in LRNR and TSLF = 2 in DANR) (Figure 4). The total dung beetle biomass varied significantly across habitats and fire histories. In LRNR, savanna habitats presented the highest dung beetle biomass (ANOVA: F = 7.6; *p* = 0.0096), except in areas with recent fires (TSLF ≤ 1 year) and two fires, where the highest biomass was recorded in edge habitats (Figure 5a,b). Across all habitats, dung beetle biomass peaked at TSLF = 6 years (Figure 5c). Across all habitats, dung beetle biomass peaked at TSLF = 6 years (Figure 5c).

The generalized linear mixed model (GLMM) results revealed a trend of increasing biomass with increasing fire occurrence and decreasing biomass with increasing TSLF (Figure 5e). At the guild level, small dwellers and large tunnellers presented the strongest responses to fire occurrence (Figure 5f), but only small dwellers were significantly affected by the TSLF, showing reduced biomass with increasing recovery time (Figure 5f). Small tunnellers exhibited differential responses, with biomass peaking in areas with four fire occurrences and a TSLF = 6 years (Figure 5f). Similarly, the biomass of the small rollers and medium tunnellers was highest at TSLF = 1 and 9 years, respectively (Figure 5f).

### 3.2. Taxonomic and Functional Dung Beetle Responses to Fire on the Riparian Forest–Savannah Transition

Species richness: Overall, habitat-specific beetle communities showed contrasting trends in abundance and richness across the fire scenarios evaluated. Forests and edges supported greater beetle abundance than savannas did, regardless of fire history. In contrast, edge and savannah habitats maintained low abundances regardless of fire history (Nprom = 43.6 and Nprom = 13.8, respectively) (Figure S2). Savanah and edge richness increased with respect to fire occurrence, except in the forest habitat, where the lowest values (Sprom = 3) occurred during the five fire events (Figure S1). Regardless of fire scenarios in the conserved area (DANR), forest habitats housed abundant dung beetle communities in contrast with edge and savanna areas. In addition, the transition from unburned forest to edge with 16 years since the last fire event and one fire occurrence had the highest abundance values (ANOVA F = 7,908; *p* = 0.001) (Figure S1). Richness remained constant in terms of fire occurrence and time since the last fire scenario, with the highest values found in the edge habitat (Sprom = 5.36).

Specifically with time since the last fire event, edge richness (Sprom = 5.3) was greater than that of forests and savannah (Sprom = 4.1 and Sprom = 2.6, respectively), except for forests 0 years after the last fire event. In contrast, the general richness trend in forests and savannahs increased with recent fires (Figure S2). Overall, species diversity was lower in sites with a TSLF = 6 years and in areas that had experienced 4–5 fire occurrences than in the other sampled sites (Figure 2c). Rare species contributed the most to savanna species composition across fire scenarios, in contrast with the general pattern of communities experiencing different fire occurrences and times since the last fire event (TSLF), which exhibited similar rare species patterns (Figure 2a). In contrast, forest habitats supported both dominant and rare species, regardless of fire history (Figure 2a–c).

Communities from unburned forest and edges housed the largest number of species, which were also present in most of the fire-affected scenarios (Figure 3a,b), except for a few species that exhibited strong habitat specialization. *Ateuchus aphoplygus*, *Canthon juvencus*, and *Onthophagus bidentatus* were more frequently found in forests, whereas *Pseudocanthon xanthurus*, *P. marginicollis*, and two species of Aphodinae (sp12 and sp14) were collected exclusively in savannah ecosystems (Figure 3c) (ANOSIM R = 0.519; *p* = 0.001).

In terms of functional diversity and composition, in some of the unburned forests, small rollers were notably abundant, whereas recent fires (0–3 years since the last fire event) had a negative impact on this guild (Figure 4). Small tunnellers were particularly abundant in forest and edge habitats with low fire occurrence rates (0–2 events) during the first three years after fire disturbances (Figure 4). In contrast, medium- and large-sized tunnellers remained stable in savannas and edges regardless of time since the last fire event. The total dung beetle biomass varied significantly across habitats and fire histories. For instance, savanna habitats presented the highest dung beetle biomass (ANOVA: F = 7.6; *p* = 0.0096), except in areas with recent fires (≤1 year) and two fires events, where the highest biomass was recorded in edge habitats (Figure 5a,b). Across all habitats, dung beetle biomass peaked at TSLF = 6 years (Figure 5c).

### 3.3. Natural Reserve Conservation Status and Fire Effects on Dung Beetle Assemblages

With respect to specific species recollected in natural reserves *Coprophanaeus gamezii*, *Ateuchus aphoplygus*, and *Ontherus appendiculatus* are unique species from the most conserved locality (DANR). *Sybalocanthon sexpilotus* and *Canthon mutabilis* were exclusive to the LRNR. Species richness and abundance declined with increasing fire frequency and shorter recovery times, particularly in the LRNR. Also, dung beetle abundance declined significantly with increasing fire frequency, with the highest abundance in unburned forests and forests with TSLF = 2 years (ANOVA F= 9.111; *p* = 0.018). In contrast, the dung beetle abundance in DANR communities decreased with increasing fire occurrence and less time since the last fire event; in contrast with edge and savanna areas, forest habitats housed abundant dung beetle communities.

In terms of beta diversity, the community differentiation between the two study sites was more pronounced, with DANR and LRNR showing significant segregation in terms of species composition (ANOSIM R = 0.105; *p* = 0.001) (Figure 3d). Additionally, species turnover analysis revealed that 68% of the total beta diversity was driven by three dominant species: *U.* cf. *brevis* (31.86%), *D. gazella* (20.05%), and *Dichotomius nisus* (16.43%) (Figure S3).

Guild composition and abundance showed contrasting results; for example, in the unburned forests of DANR, small rollers were notably abundant, whereas in LRNR, recent fires (TSLF = 0–3 years) had a negative impact on this guild (Figure 4). Small tunnellers’ abundance decreased significantly with respect to recent fires in LRNR compared to DANR, where a notable effect of the cumulative occurrence of fires was observed (Figure 4). Although no effects on the richness of small dwellers were observed, a clear effect of fire occurrence on the abundance of this guild was recorded regardless of the type of nature reserve. Medium- and large-sized tunnellers showed contrasting results in relation to the conservation status of habitats. In DANR, the abundance and richness of medium-sized tunnellers remained stable despite the occurrence of fires, while in LRNR, these variables decreased in response to the accumulation of fires (Figure 4).

Conversely, large tunnellers’ abundance and richness were stable in DANR regardless of time since the last fire event and occurrence. In LRNR, this guild showed the lowest values in both unburned and burned habitats. The highest total dung beetle biomass was found in open habitats in DANR (ANOVA: F = 7.6; *p* = 0.0096), while in LRNR, it was observed in areas without recent fires (Figure 5). In DANR, the biomass patterns showed a clear pattern of growth with respect to recent and more frequent fires (ANOVA: F = 16.84; *p* = 0.0001), with the exception of the high values occurring in habitats 6 years after the last fire event (Figure 5).

## 4. Discussion

This study provides the first comprehensive assessment of the effects of fire on dung beetle assemblages within the riparian forest–savanna transitions of the Colombian Orinoquía. While a core set of species characterized beetle communities in both reserves, differences in habitat conservation status influenced the overall ecological dynamics. Fire occurrence and time since the last fire event had differential effects on dung beetle communities, with recent fires and less-conserved habitats exhibiting the strongest negative impacts on taxonomic and functional diversity.

### 4.1. How Do Dung Beetle Composition, Richness, Abundance (Taxonomic and Functional), and Biomass Vary Under Different Fire Scenarios?

One of the most striking findings was the dominance of *Uroxys* cf. *brevis*, an indicator species of high fire recurrence. However, despite their adaptability to transitional environments, their abundance has declined significantly in response to recent fire events, particularly in less-conserved habitats. This result aligns with studies demonstrating that small-sized dung beetles, particularly within the *Uroxys* genus, persist in undisturbed ecosystems where stable resource availability supports their populations [66,67]. The frequent presence of *D. gazella* in fire-prone areas suggests that its competitive advantage may allow it to outcompete native species, particularly those sensitive to thermal fluctuations and microhabitat changes. This species has successfully colonized diverse ecosystems worldwide because of its high dispersal ability, short generation time, and broad tolerance to disturbed environments [68,69].

Interestingly, we observed an abundance recovery trend at TSLF = 2 years in LRNR and at TSLF = 6 and 16 years where dung beetle populations reached levels similar to those in unburned habitats. However, this recovery was driven by dominant species, such as *Uroxys* cf. *brevis* and *D. nisus*, rather than a complete restoration of the prefire community composition. This suggests that postfire beetle communities undergo a restructuring process, where generalist and disturbance-tolerant species recolonize burned areas, whereas more specialized species struggle to reestablish burned areas. This pattern is commonly observed in degraded landscapes, where functional homogenization occurs due to the loss of ecologically sensitive species [55,70,71].

While local dynamics associated with the effects of fire marked clear trends in fire occurrences and recovery times, a pattern of species nesting was observed. The composition of dung beetle communities in unburned habitats resulted in assemblages with different structures depending on the specific fire scenario. In this sense, unburned habitats potentially acted as the main reservoir of species that, depending on their adaptations, managed to disperse in response to fire disturbance. In some fire-impacted tropical forest and savanna landscapes, the proximity of unburned areas was crucial for the movement of beetle populations to and from burned sites [72,73,74].

Dung beetle guilds exhibited differential responses to fire history, with small tunnellers dominating in terms of abundance and small dwellers showing the highest richness. While small tunnellers primarily occupied unburned forests and edge habitats, small dwellers were more abundant in edges and savannas affected by recent fires. Fire scenarios characterized by high frequency (4–5 fire occurrences) and recent burns (TSLF = 0–3 years) negatively affected small tunnellers and small rollers, significantly reducing their abundance and richness. Our findings aligned with those of previous studies showing that disturbed environments tend to be dominated by small- and medium-sized burrowing beetles, compensating for the absence of larger species [52,75,76]. Similar trends have been reported in fire-prone ecosystems, where small species such as *Canthon curvodilatatus* [39], *Ateuchus histeroides* [77,78], *Onthophagus bidentatus* [79], and beetles of the subfamily Aphodiinae [40,78] demonstrate resilience. The resilience of small dwellers is likely due to their low metabolic rates and ability to utilize lower-quality feces in fire-impacted areas, where resource availability for other beetle groups is diminished [80,81].

Resource supply and quality play crucial roles in maintaining functional diversity and ecosystem processes. In our study, dung availability was largely determined by the presence of herbivorous mammals, including white-tailed deer (*Odocoileus cariacou*), capybaras (*Hydrochoerus hydrochaeris*), and cattle at savannah and forest edges, followed by tapirs (*Tapirus terrestris*), picures (*Dasyprocta fulginosa*), and peccaries (*Pecari tajacu*). These mammals were observed more frequently in conserved habitats, where stable fecal resources likely contributed to greater functional diversity.

Larger dung beetle guilds, which have higher food and habitat requirements, are more sensitive to fire disturbances, as shown by the negative effects of fire on large burrowing beetles and dwellers [82,83]. The impact of fire on large-bodied dung beetles has been well documented, with studies showing that elevated temperatures and feces desiccation limit their survival and reproductive success [32,84]. Large dwellers, such as *Eurysternus* spp., were absent from all fire-affected areas, suggesting that fire-related habitat changes severely restrict their persistence [39,40]. These trends highlight the role of functional redundancy in postfire recovery, with small guilds compensating for the loss of large-bodied beetles. However, such shifts may compromise key ecosystem functions, such as deep soil bioturbation, large-seed dispersal, and high-volume dung removal, which are driven primarily by larger beetles [30,85,86].

### 4.2. How Do Specific Habitats of the Riparian Forest–Savannah Transition Influence Responses to Fire at the Taxonomic and Functional Levels?

Forest edges played an important role in maintaining the diversity of dung beetles in the fire scenarios evaluated, particularly in the scenarios of the greatest occurrence of recent fires. Dung beetle species richness often decreases with increasing environmental harshness, as extreme temperatures and resource scarcity limit the survival of specialized species [87,88]. Notably, edge habitats presented the highest species richness across all fire scenarios. This highlights the role of ecotones as biodiversity hotspots, supporting both forest-adapted and savanna-adapted species. While forest habitats support both dominant and rare species, savanna assemblages are structured primarily by rare species, suggesting that savannah may act as transient habitats rather than stable refugia for dung beetles. In these habitats in particular, the ability to access a resource supply in the postfire period is often determined by rapid access to olfactory cues associated with the volatiles produced by dung [39,89]. The ability to move is recognized as an adaptation that can determine the survival of fire-resistant beetle species. In this sense, rapid flight or locomotion allows them to evade both flames and to efficiently access available feces [37,38].

The composition of unburned forest communities plays a crucial role in determining the structure of postfire assemblages. These intact habitats likely acted as species reservoirs, with dung beetle dispersal shaping recolonization patterns at burned sites. Similar postfire recovery patterns have been documented in tropical landscapes, where the presence of nearby undisturbed areas enables species persistence despite periodic disturbances [72,74]. For species tolerant of these transitions, edges offer new microhabitats and resources that can benefit their fitness [87]. For example, in natural forest–grassland ecotones, various dung beetle species can show greater abundance both within forests and grasslands; this highlights the role of habitat dissimilarity as an ecological determinant [90,91]. In this context, edge habitats may serve as critical ecological corridors, facilitating movement between forest patches and allowing species to track resource availability over time [87].

Dung beetle guilds exhibited differential responses to fire history, with small tunnellers dominating in terms of abundance and small dwellers showing the highest richness. While small tunnellers primarily occupied unburned forests and edge habitats, small dwellers were more abundant in edges and savannas affected by recent fires. Fire scenarios characterized by high frequency (4–5 fire occurrences) and recent burns (TSLF = 0–3 years) negatively affected small tunnellers and small rollers, significantly reducing their abundance and richness. The responses of guilds to fire are linked to reproductive strategies, body size, and resource use. In tropical ecosystems, disturbance events commonly filter dung beetle communities, favoring species with short life cycles and high reproductive rates [92,93]. Smaller beetles have lower nutritional demands and greater thermal tolerance, allowing them to persist in disturbed environments where large-bodied species decline [82,94]. Their perch-type foraging behavior further enhances their competitive advantage in altered landscapes [95,96].

Small rollers were disproportionately affected by recent fire events, particularly in forest habitats. Many small rollers rely on canopy-dwelling mammals such as primates as dung sources and may be more vulnerable to fire-induced declines in these host populations [97,98,99]. *Canthon juvencus*, which was restricted to unburned forests in this study, has been linked to primate dung availability [100,101]. Since fire significantly alters vegetation structure, resource distribution, and mammal community composition [4], it is likely to reduce the availability of dung resources for forest-dependent rollers, contributing to their postfire decline.

Overall, fire had a positive effect on dung beetle biomass, particularly in savanna habitats where postfire resource conditions facilitated dung beetle activity. In some tropical ecosystems, fires can increase dung availability by concentrating herbivores in newly burned areas, where fresh vegetation regrowth attracts grazing species [38,39]. In this study, the high biomass observed in fire-affected savannas was influenced by the movement of beetles from unburned areas, as well as the presence of cattle, deer, and other large mammals maintaining fecal resource stability [102,103].

The observed compensatory effect between abundance and biomass suggests that in functionally reduced assemblages, a few highly abundant species may dominate biomass contributions. A clear example of this pattern was *Dichotomius nisus*, an indicator species of high fire occurrence, which exhibited high biomass accumulation in savanna habitats with 3–4 fire events. This dominance suggests that fire-adapted species may drive postfire functional restructuring and maintain ecosystem processes despite community simplification. Furthermore, the biomass peaks observed in small rollers, medium-sized tunnellers, and small dwellers in unburned and recovering habitats reinforce the idea that fire history shapes the distribution of functional traits, influencing long-term ecosystem dynamics.

### 4.3. What Is the Relationship Between Conservation Status and Fire Effects on Dung Beetle Assemblages?

In general, the abundance of dung beetle communities was a variable sensitive to fire in both conservation scenarios. Particularly the lowest generalized values were observed for the habitats with recent fires, with fire occurrences from 2 to 3 in LRNR and 3 to 4 in DANR. Dung beetle assemblages have been recognized as indicators of ecosystem health, mainly in tropical ecosystems, because their abundance and richness are sensitive to anthropogenic disturbances [82,104]. This response is directly and indirectly related to abiotic and biotic alterations of the habitat of these organisms, with mainly the change in vegetation cover impacting temperature and humidity conditions [105,106], which affects the quality and food supply and, consequently, the fitness of dung beetles [107,108,109]. The dung beetle richness and abundance declines presented here are consistent with studies demonstrating that dung beetle assemblages function as indicators of ecosystem health, responding strongly to disturbances such as fires, deforestation, and habitat fragmentation [83,109]. Fire-induced habitat alterations disrupt microclimatic conditions, reduce resource availability, and affect mammal populations, indirectly impacting dung beetle survival [110,111].

In fire-sensitive ecosystems, dung beetles often migrate away from burned areas to seek more favorable conditions, further exacerbating local declines in abundance and diversity [72,73,74]. Ecosystems with fewer anthropogenic disturbance factors provide stable resources for dung beetle assemblages, where the vegetation structure allows the permanence of medium-sized mammals and provides microclimatic stability, primarily for stenotypic species [112,113,114,115]. This factor is even more important in the context of fire scenarios, where conserved habitats maintain ecological stability that positively impacts the restructuring processes of biological communities in the postfire environment [4,26,28,116,117]. In this study, we found a general nesting pattern in which most species present in the non-conserved area were in the reserve assemblages with a stable conservation status. This landscape-level scenario, where core beetle assemblages exist, allows for the gradual dispersal of populations to new areas undergoing recovery [118,119,120]. At this scale, the ecological dynamics of dung beetle metacommunities generate resilience processes with respect to fire-induced disturbances; an example of this was recorded in tropical savannah communities with fire occurrences in Campos Rupestres in Brazil [39].

At the functional level, a general pattern was observed in which small tunnellers remained relatively stable across the different fire scenarios. In contrast, large tunnellers beetles presented site-specific responses, which declined in LRNR but partially recovered in DANR, where the habitat conditions were relatively stable. In these assemblages with functional redundancy based on small-sized beetles, some specific functions can be maintained; however, it has been recognized that the absence of or decrease in the activity of large-sized guilds impacts functions such as the removal of excrement and the dispersal of large seeds [52,95]. In tropical rainforests in Brazil impacted by the combination of anthropogenic stressors and the presence of fires, decreases in the above-mentioned specific dung beetle functions were observed, with these decreases arising from disturbance effects on specific large beetle guilds [43].

## 5. Conclusions

This study provides a comprehensive evaluation of how fire occurrence and time since the last fire event (TSLF) shape dung beetle assemblages in the riparian forest–savanna transitions of the Colombian Orinoquía. Our findings highlight that recent fires (TSLF ≤ 3 years) and high fire frequency (4–5 occurrences) negatively impact species richness and abundance, affecting both taxonomic and functional diversity. Although forests supported the highest diversity, savannas were dominated by disturbance-tolerant species. Edge habitats play a key role as biodiversity reservoirs, housing species from both ecosystems, and serving as transition zones for postfire recovery. Notably, fire-sensitive species were restricted primarily to unburned forests, whereas disturbance-tolerant species, such as *D. gazella*, thrived in frequently burned areas.

At the functional level, small tunnellers and small dwellers exhibited high resilience, maintaining stable populations across fire scenarios. In contrast, small rollers have declined in response to recent fires due to reductions in mammal species that provide key dung resources in forest ecosystems. Large-bodied beetles were the most negatively impacted by fire, with large burrowing beetles and large dwellers disappearing from burned areas. These declines could have significant consequences for ecosystem functions, such as deep soil bioturbation, nutrient cycling, and large-seed dispersal. Despite reductions in species diversity, biomass increased in fire-affected areas, particularly in savannas, where postfire resource conditions favor species with high dispersal abilities. The dominance of a few highly abundant species, such as *D. nisus*, suggests a compensatory role for functionally redundant species in maintaining biomass under fire-prone conditions. However, this shift may lead to functional homogenization, where ecosystem processes increasingly rely on disturbance-tolerant species rather than diverse functional assemblages.

The findings of this study have important implications for fire management and biodiversity conservation in the Orinoquía region and other fire-prone tropical ecosystems. The conservation of unburned forests and edge habitats is essential for maintaining dung beetle diversity and ensuring postfire recovery. Reducing fire frequency in critical habitats, particularly riparian forests, could help mitigate biodiversity loss and preserve key ecosystem functions. Furthermore, given their sensitivity to environmental changes, monitoring dung beetle assemblages can serve as an effective bioindicator tool for assessing fire impacts and guiding conservation strategies.

This study underscores the complex interactions among fire regimes, habitat structure, and dung beetle functional diversity, emphasizing the need for integrated fire management strategies that balance disturbance dynamics with biodiversity conservation. Future research should focus on long-term monitoring of postfire recovery in dung beetle communities, examining species-specific physiological adaptations to fire, and understanding the cascading effects of fire-induced mammal decline on dung beetle functional roles. By integrating fire ecology with conservation planning, it is possible to mitigate fire-related biodiversity losses and develop sustainable land management practices that preserve ecosystem integrity in fire-sensitive regions.

## Data Availability

The data presented in this study are available upon request from the corresponding author.

## Supplementary Materials

The following supporting information can be downloaded at https://www.mdpi.com/article/10.3390/biology14040423/s1. Figure S1: Richness and abundance of dung beetle species in fire scenarios. LRNR: abundance according to (a) fire occurrence and (b) TSLF; richness according to fire occurrence (e) and TSLF (f). DANR: abundance according to (c) fire occurrence and (d) TSLF; richness according to fire occurrence (g) and TSLF (h); black: forest, cyan: edge, and blue: savannah. Table S1: Total species collected in DANR and LANR. TSLF: time since the last fire event. F: forest, E: edge and S: savannah. Send: small endocoprid, STun: small tunneller, SRol: small roller, Btun: big tunneller, and MTun: medium tunneller. Table S2: Indicator species in the fire scenarios.
