# Correction: Moreno-Fonseca et al. Postfire Scenarios Shape Dung Beetle Communities in the Orinoquía Riparian Forest–Savannah Transition. *Biology* 2025, *14*, 423

**DOI:** 10.3390/biology14070789

**Published:** 2025-06-30

**Authors:** Carlos Julián Moreno-Fonseca, Jorge Ari Noriega, Walter Garcia-Suabita, Dolors Armenteras-Pascual

**Affiliations:** 1 Grupo de Investigación en Ecología del Paisaje y Modelación de Ecosistemas-ECOLMOD, Departamento de Biología, Universidad Nacional de Colombia, Bogotá 111311, Colombia; walter.suabita@gmail.com (W.G.-S.); darmenterasp@unal.edu.co (D.A.-P.); 2 Grupo de Agua, Salud y Ambiente, Programa de Ingeniería Ambiental, Facultad de Ingeniería, Universidad El Bosque, Bogotá 111321, Colombia; jnorieg@hotmail.com

## Addition of an Author

Jorge Ari Noriega was not included as an author in the original publication [1]. The corrected Author Contributions statement appears here. The ‘Acknowledgments’ section was updated to read: “We would like to acknowledge Santiago Rodriguez for field and laboratory assistance; and Doña Ana Natural Reserve and Los Robles Natural Reserve crew for field assistance and support.”

The authors state that the scientific conclusions are unaffected. This correction was approved by the Academic Editor. The original publication has also been updated.

**Author Contributions:** Conceptualization, C.J.M.-F. and D.A.-P.; data curation, W.G.-S. and J.A.N.; formal analysis, C.J.M.-F. and W.G.-S.; investigation, C.J.M.-F., J.A.N., D.A.-P., and W.G.-S.; methodology, C.J.M.-F. and J.A.N.; resources C.J.M.-F. and J.A.N.; supervision, J.A.N. and D.A.-P.; visualization, C.J.M.-F. and W.G.-S.; writing—original draft, C.J.M.-F.; writing—review and editing, C.J.M.-F. and D.A.-P. All authors have read and agreed to the published version of this manuscript.
