# Supplementary material for: Postfire Scenarios Shape Dung Beetle Communities in the Orinoquía Riparian Forest–Savannah Transition
Source: Biology (Basel). 2025 Apr 15;14(4):423. doi: 10.3390/biology14040423 (PMC12024859; doi:10.3390/biology14040423)
Supplement: Supplementary file 1 [file biology-14-00423-s001.zip › biology-3527331-supplementary.pdf]

Supplementary Material

Table S1. Total species recollected in DANR and LANR. TSLF: time since last fire event. F: forest, E: edge, and S: savannah. Send: small endocoprid, STun: small tunneller, SRol: small roller, Btun: big tunneller and MTun: medium tunneller.

				**Fire occurrence**		**3**			**1**			**2**			**4**			**0**			**1**		
	DANR			**TSLF**		**2**			**16**			**3**			**6**			**22**			**10**		
				**Sample point**		**1**			**2**			**3**			**4**			**5**			**6**		
**#**	**Subfamily**	**Tribe**	**Genus**	**Species**	**Guild**	**F**	**E**	**S**	**F**	**E**	**S**	**F**	**E**	**S**	**F**	**E**	**S**	**F**	**E**	**S**	**F**	**E**	**S**
1	Aphodiinae			Sp. 1	SEnd	11	4	0	0	8	0	1	1	0	1	8	1	0	0	0	1	1	0
2	Aphodiinae			Sp. 2	SEnd	2	4	0	0	6	74	2	5	2	0	0	0	2	4	0	2	8	0
3	Aphodiinae			Sp. 3	SEnd	11	123	39	0	51	74	3	281	81	7	7	3	0	6	38	75	95	171
4	Aphodiinae			Sp. 4	SEnd	0	0	0	0	0	0	0	0	0	1	1	0	0	0	1	0	0	0
6	Aphodiinae			Sp. 6	SEnd	0	1	2	0	0	1	0	0	0	0	0	0	0	0	0	0	0	3
7	Aphodiinae			Sp. 7	SEnd	0	1	0	3	27	2	0	4	0	0	1	0	3	5	1	3	1	3
9	Aphodiinae			Sp. 9	SEnd	0	4	2	2	7	1	4	18	1	0	1	0	4	29	5	1	9	1
10	Aphodiinae			Sp. 10	SEnd	0	0	0	0	0	0	0	0	0	0	0	0	0	0	0	0	1	0
11	Aphodiinae			Sp. 11	SEnd	0	0	0	0	0	0	0	0	0	0	0	0	0	0	1	0	1	2
12	Aphodiinae			Sp. 12	SEnd	0	0	0	0	0	1	0	0	0	0	0	0	0	0	39	0	0	0
13	Aphodiinae			Sp. 13	SEnd	0	0	0	0	1	1	0	0	0	0	0	0	0	0	0	0	0	0
14	Aphodiinae			Sp. 14	SEnd	0	0	2	0	0	0	0	0	0	0	0	0	0	0	0	0	0	0
15	Scarabaeinae	Ateuchini	*Ateuchus*	*aphoplygus*	SEnd	0	0	0	5	5	0	0	0	0	0	0	0	1	2	0	1	3	0
16			*Ateuchus*	sp. 1	SEnd	0	0	0	1	1	7	0	0	0	0	0	0	0	0	0	0	0	0

Continuation of Table S1. Total species recollected in DANR and LANR. TSLF: time since last fire event. F: forest, E: edge, and S: savannah. Send: small endocoprid, STun: small tunneller, SRol: small roller, Btun: big tunneller and MTun: medium tunneller.

	DANR																						
				**Fire occurrence**			**3**			**1**			**2**			**4**			**0**			**1**	
				**TSLF**			**2**			**16**			**3**			**6**			**22**			**10**	
				**Sample point**			**1**			**2**			**3**			**4**			**5**			**6**	
**#**	**Subfamily**	**Tribe**	**Genus**	**Species**	**Guild**	**F**	**E**	**S**	**F**	**E**	**S**	**F**	**E**	**S**	**F**	**E**	**S**	**F**	**E**	**S**	**F**	**E**	**S**
17			*Uroxys*	sp. 1	SEnd	345	192	3	1445	773	12	1475	198	0	5	1	0	1056	630	0	1256	403	5
18		Coprini	*Ontherus*	cf. *appendiculatus*	STun	0	10	4	0	3	5	2	7	4	9	11	0	0	8	19	0	10	14
19		Dichotomiini	*Canthidium*	sp. 1	STun	0	0	0	0	0	0	0	0	0	0	1	0	0	0	0	0	0	0
20			*Canthidium*	sp. 2	STun	0	2	0	0	1	0	0	4	1	0	1	0	0	0	0	0	3	0
21			*Canthidium*	sp. 3	STun	1	0	0	0	0	0	0	0	1	0	0	0	0	1	0	0	0	0
22			*Onthophagus*	*bidentatus*	STun	1	0	0	0	0	0	3	0	0	0	1	0	0	0	0	0	0	0
23			*Onthophagus*	*marginicollis*	STun	0	0	1	0	0	50	0	0	0	0	0	0	0	0	0	0	0	0
24			*Dichotomius*	*nisus*	Btun	55	125	141	39	51	50	35	107	40	110	90	74	57	93	224	7	64	106
34		Phanaeini	*Coprophanaeus*	*gamezi*	Btun	0	0	0	0	0	0	0	0	0	0	0	0	0	0	1	0	0	0
25		Deltochiliini	*Canthon*	*juvencus*	SRol	0	6	0	2	0	1	4	0	0	0	0	0	47	5	0	2	1	0
27			*Canthon*	sp. 1	SRol	0	0	0	0	0	1	0	0	0	0	1	0	0	0	1	0	0	1
28			*Canthon*	sp. 2	SRol	0	0	0	0	0	1	0	0	0	1	1	0	0	0	2	0	0	0
30			*Pseudocanthon*	cf. *felix*	SRol	0	0	0	0	0	2	0	0	0	0	0	0	0	0	1	0	0	0
31			*Pseudocanthon*	sp. 1	SRol	9	3	2	0	3	14	0	4	0	0	2	0	0	2	0	0	1	1
33		Onthophagini	*Digitonthophagus*	*gazella*	Mtun	0	3	23	0	0	13	0	0	5	5	13	36	0	0	26	0	0	15

Continuation of Table S1. Total species recollected in DANR and LANR. TSLF: time since last fire event. F: forest, E: edge, and S: savannah. Send: small endocoprid, STun: small tunneller, SRol: small roller, Btun: big tunneller and MTun: medium tunneller.

					**Fire occurrence**	**1**			**0**			**1**			**2**			**3**			**5**		
	LRNR				**TSLF**	**9**			**22**			**1**			**0**			**3**			**2**		
					**Sample Point**	**1**			**2**			**3**			**4**			**5**			**6**		
#	**Subfamily**	**Tribe**	**Genus**	**Species**	**Guild**	**F**	**E**	**S**	**F**	**E**	**S**	**F**	**E**	**S**	**F**	**E**	**S**	**F**	**E**	**S**	**F**	**E**	**S**
1	Aphodiinae			Sp.1	SEnd	**15**	**5**	**0**	**0**	**7**	**0**	**4**	**13**	**0**	**50**	**1**	**1**	**38**	**27**	**3**	5	16	5
2	Aphodiinae			Sp. 2	SEnd	1	2	0	0	0	0	0	0	1	3	0	0	0	0	0	0	0	0
3	Aphodiinae			Sp. 3	SEnd	0	1	0	0	1	0	0	1	1	2	11	1	2	9	4	0	5	1
4	Aphodiinae			Sp. 4	SEnd	0	1	0	0	21	1	0	3	0	0	8	2	0	14	1	0	1	2
5	Aphodiinae			Sp. 5	SEnd	0	0	0	0	0	0	0	0	0	0	0	0	0	2	0	0	2	5
6	Aphodiinae			Sp. 6	SEnd	0	0	0	0	0	0	0	0	0	0	0	0	0	1	0	0	1	0
7	Aphodiinae			Sp. 7	SEnd	0	1	0	0	0	0	0	0	0	0	1	1	7	0	0	0	0	0
8	Aphodiinae			Sp. 8	SEnd	0	0	0	0	0	0	0	0	0	0	0	0	0	2	0	0	0	0
12	Aphodiinae			Sp. 12	SEnd	0	0	0	0	0	0	0	0	0	0	0	2	0	0	0	0	0	0
14	Aphodiinae			Sp. 14	SEnd	0	0	0	0	0	0	0	0	0	0	0	0	0	0	0	4495	0	1
17	Scarabaeinae	Ateuchini	*Uroxys*	sp. 1	SSca	974	124	0	3253	136	0	1466	226	5	111	5	0	972	77	0	4496	577	5
18		Coprini	*Ontherus*	cf. *appendiculatus*	SSca	1	0	0	1	0	0	0	2	0	1	1	0	1	0	0	1	0	0
19		Dichotomiini	*Canthidium*	sp. 1	SSca	0	0	0	0	0	1	0	0	0	0	0	0	0	0	0	0	0	0

Continuation of Table S1. Total species recollected in DANR and LANR. TSLF: time since last fire event. F: forest, E: edge, and S: savannah. Send: small endocoprid, STun: small tunneller, SRol: small roller, Btun: big tunneller and MTun: medium tunneller.

LRNR					**Fire occurrence**	**1**			**0**			**1**			**2**			**3**			**5**		
					**TSLF**	**9**			**22**			**1**			**0**			**3**			**2**		
					**Sample Point**	**1**			**2**			**3**			**4**			**5**			**6**		
**#**	**Subfamily**	**Tribe**	**Genus**	**Species**	**Guild**	**F**	**E**	**S**	**F**	**E**	**S**	**F**	**E**	**S**	**F**	**E**	**S**	**F**	**E**	**S**	**F**	**E**	**S**
20				sp. 2	SSca	0	0	0	0	0	0	1	0	0	0	0	0	0	0	0	0	0	0
21				sp. 3	SSca	0	0	0	0	0	0	0	0	0	0	0	0	0	0	0	6	0	0
22			*Onthophagus*	*bidentatus*	SSca	26	13	0	12	2	0	10	8	0	0	0	0	5	9	1	6	1	0
24			*Dichotomius*	*nisus*	BSca	0	2	5	0	6	8	0	3	3	6	11	27	4	8	35	468	2	16
25		Deltochiliini	*Canthon*	*juvencus*	SRol	209	29	0	129	27	0	203	32	0	0	0	0	12	1	0	468	31	0
26				*mutabilis*	SRol	0	0	0	0	0	0	0	0	0	0	0	1	0	0	0	0	0	0
27				sp. 1	SRol	1	0	0	0	0	0	0	0	0	2	4	2	0	1	0	0	1	0
28				sp. 2	SRol	0	0	0	0	0	0	0	0	0	0	0	0	0	0	0	0	0	1
30			*Pseudocanthon*	cf. *felix*	SRol	0	1	2	0	2	2	0	0	1	0	1	0	0	1	0	0	0	3
29				cf. xanthurus	SRol	0	0	1	0	1	0	0	0	2	0	0	7	0	0	11	0	2	16
31				sp. 1	SRol	1	1	0	1	1	1	2	1	0	4	0	0	1	3	0	0	10	0
32			*Scybalocanthon*	*sexpilotus*	MRol	0	0	0	0	0	0	1	0	0	0	0	0	0	0	0	0	0	0
33		Onthophagini	*Digitonthophagus*	*gazella*	MSca	0	3	46	0	8	41	0	1	46	12	23	76	4	6	60	0	10	42

Figure S1. Richness and abundance of dung beetle species in different fire scenarios. LRNR: abundance according to (a) fire occurrence, and (b) TSLF; richness according to fire occurrence (e), and TSLF (f). DANR: abundance according to (c) fire occurrence, and (d) TSLF; richness according to fire occurrence (g), and TSLF (h). Black: forest, cyan: edge, and blue: savannah.

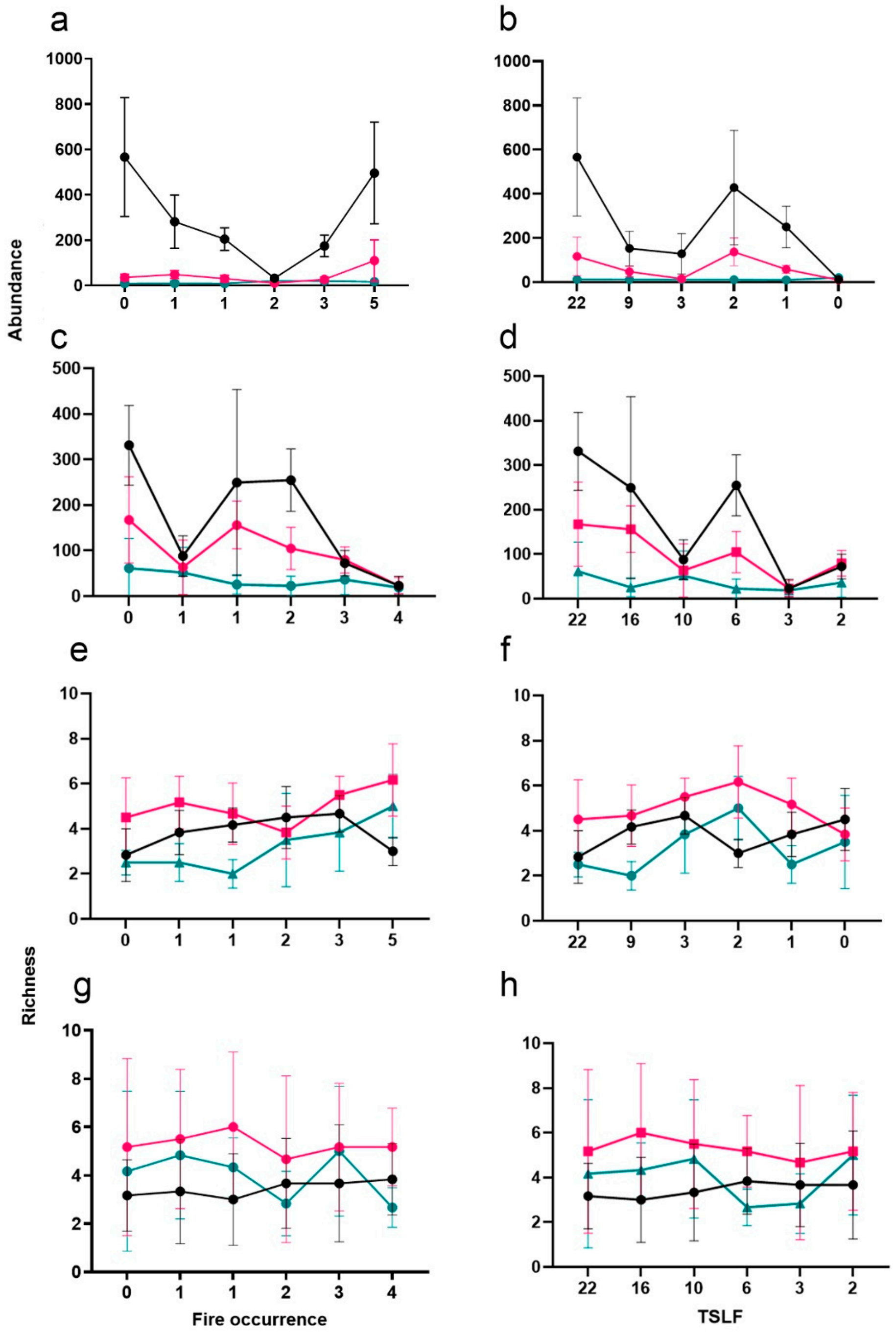

Table S2. Indicator species in fire scenarios.

Variable	Species	IndValA	IndValb	Stat	p.value
Fire Occurrence 5	*Uroxys cf. brevis*	0,4624	0,8333	0,621	0,001
Fire Occurrence 4	*Dichotomius.nisus*	0,3166	0,8333	0,514	0,009
TSLF 0	*Digitonthophagus gazella*	0,2754	0,8333	0,479	0,003
